# Maximizing Deep Lung Deposition in Healthy and Fibrotic Subjects During Jet Nebulization

**DOI:** 10.1089/jamp.2019.1552

**Published:** 2020-04-02

**Authors:** Joshua Samuel, Gerald C. Smaldone

**Affiliations:** Pulmonary, Critical Care and Sleep Division, Department of Medicine, State University of New York at Stony Brook, Stony Brook, New York.

**Keywords:** aerosol, human, inhaled interferon-γ, nebulizer, scintigraphy

## Abstract

***Background:*** In volunteers with idiopathic pulmonary fibrosis (IPF), inhaled Interferon-γ (IFN-γ) is safe and may improve pulmonary function. However, coughing, associated with upper airway deposition, is often reported. To address this problem, a small-particle, breath-enhanced jet nebulizer (i-NEB Mini; InspiRx, Inc., Somerset, NJ) was developed. Using gamma scintigraphy, this device was tested in healthy individuals and subjects with IPF to determine efficiency and regional deposition in lung and airways.

***Methods:*** Four healthy individuals and nine subjects with IPF were enrolled. The nebulizer was filled with 2 mL of saline with ^99m^ Tc bound to diethylenetriaminepentaacetic acid (DTPA) powered continuously with 3.4 L/min of compressed air. Mass median aerodynamic diameter (MMAD) was measured by cascade impactor. To maximize deposition in alveoli, inspiratory flow was limited by an inspiratory resistance incorporated into the nebulizer, resulting in a deep inspiration ∼6 seconds. The treatment was run to completion (10 minutes), and each subject underwent deposition imaging. Mass balance and regions of interest determined upper airway (measured by calibrated stomach activity) and regional lung deposition as a percent of pretreatment nebulizer charge.

***Results:*** Subjects tolerated the device with no complaints. MMAD (mean [geometric standard deviation]) = 1.04 [1.92] μm. Lung deposition (mean ± standard error, % nebulizer charge) in healthy subjects was 26.2% ± 1.83 and in IPF individuals 23.4% ± 1.60 (*p* = 0.414). Upper airway deposition was 1.4% ± 0.83 and 2.3% ± 0.48, respectively (*p* = 0.351), and 20.1% was lost during expiration. Central/Peripheral ratios were consistent in both groups, showing high peripheral deposition (1.32 ± 0.050, vs. 1.28 ± 0.046, *p* = 0.912).

***Conclusion:*** The i-NEB Mini jet nebulizer with breath enhancement produced small particles, resulting in minimal upper airway deposition. Using slow and deep breathing, more than half of the emitted dose deposited in the peripheral lung in normal subjects and individuals with IPF. These data indicate that, for future clinical trials, controlled lung doses of small particles, designed to avoid coughing, are possible even in subjects with advanced disease.

## Introduction

Idiopathic Pulmonary Fibrosis (IPF) is a fatal, progressive interstitial lung disease with limited treatment options, no cure, and a median survival of 2–3 years from the time of diagnosis.^(1)^ Approved systemic therapy with oral nintedanib or pirfenidone slows advancement of the disease, but more effective treatments with fewer side effects are needed.^(2,3)^ The State University of New York at Stony Brook and New York University are jointly developing inhaled interferon-gamma (IFN-γ), an endogenously produced cytokine with antiproliferative, antifibrotic, and anti-inflammatory properties^(4)^ as a potential treatment targeted directly to the lung. Diaz et al., in a 2-year safety study, reported that inhaled therapy with IFN-γ had one reported side effect, coughing during inhalation.^(5)^ Their data suggested that in these patients, cough might be enhanced by several factors, including particles that deposited in central and upper airways and, mannitol, a necessary sugar needed for formulation stability. As part of formulation development for future studies using inhaled IFN-γ, Kanth et al. delivered test aerosols of mannitol to volunteers with IPF and reported that coughing during inhalation could be prevented if the mannitol aerosols were free of large particles.^(6)^ Their data provide a target aerosol distribution for production devices that might be used in future clinical trials.

In addition to the distribution of inhaled particles and sites of deposition, the actual dose of drug to the lungs must be known and controlled for any clinical trial. There are limited data describing aerosol deposition in subjects with pulmonary fibrosis. Diaz et al. with a slow and deep pattern of breathing and mesh technology succeeded in delivering 65% of the nebulizer charge to the lungs of restricted subjects. The device used in that study, the I-neb (Philips/Respironics, Parsippany, NJ), produced relatively large polydisperse aerosols, possibly contributing to coughing during inhalation. For future clinical studies of inhaled IFN-γ formulations, to avoid cough, aerosol delivery may best be focused on peripheral airways and alveoli. Utilizing small particles to avoid coughing may reduce drug delivery, however, because small particles tend to be exhaled during tidal breathing. These factors can confound dose estimates to the target organ in designing clinical trials.

This article tests a proprietary jet nebulizer designed to produce an aerosol of small particles, identical to the distribution reported by Kanth et al. In addition, the device incorporates an inspiratory orifice designed to limit inspiratory flow to facilitate a slow and deep breathing pattern. The goal of the study was to quantify the magnitude and reproducibility of peripheral lung deposition in a population of individuals with lung function ranging from normal to clinically relevant IPF. With the device behavior quantified, and the degree of reproducibility confirmed, future formulations of IFN-γ can be developed containing sufficient drug to allow clinical efficacy trials.

## Methods

### *In vitro* studies

**FIG. 1.**
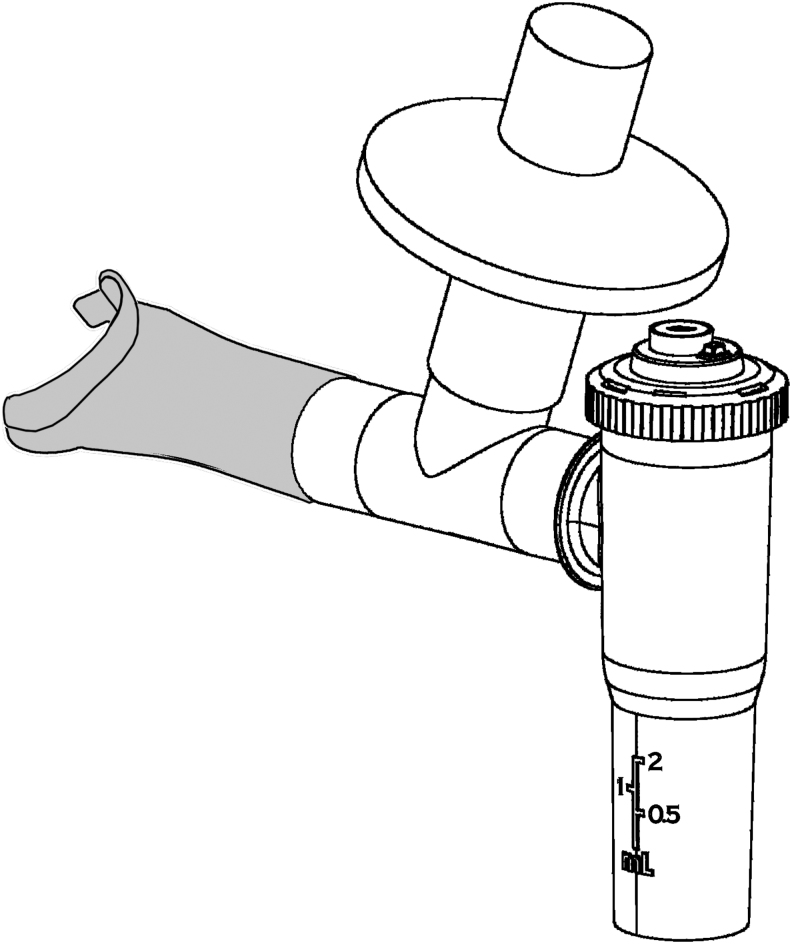
The i-NEB Mini nebulizer, fitted with a mouthpiece and a filter holder for capturing exhaled particles. The orifice limiting inspiratory flow is shown on the nebulizer cap. A one-way valve (not shown) is fitted in the cap below the orifice.

The nebulizer, InspiRx i-NEB Mini (InspiRx Inc., Somerset, NJ) (Fig. 1) is a breath-enhanced jet nebulizer powered by a portable compressor (Traveler; DiVilbiss, Port Washington, NY) at 3.4 L/min, 16 pounds per square inch (PSI), measured under load connected to the nebulizer. Inspiratory gases enter the top of the nebulizer via a one-way valve that directs patient flow into the nebulizer for breath enhancement. The nebulizer top also contains an orifice (2.30 mm) in line with the one-way valve designed to limit inspiratory flow. The diameter of the orifice was defined in separate experiments in human volunteers to provide a deep inspiration over ∼6 seconds. Once inspiration begins, inspiratory flow is fixed at about 0.5 L/s no matter how much effort is used by the subject. *In vitro* bench studies were carried out to confirm the behavior of the nebulizer design by using this breathing pattern before testing in humans.

**FIG. 2.**
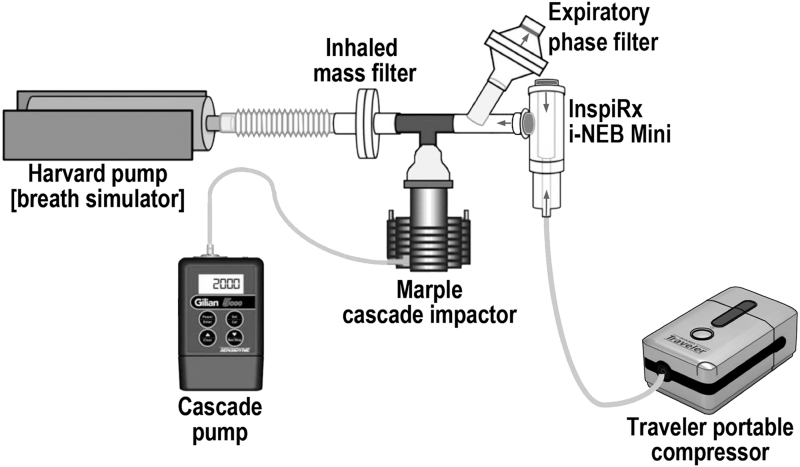
*In vitro* experimental setup for measuring nebulizer function and particle distribution during simulated patient breathing.

Five examples of the nebulizer used for human subject testing were tested on the bench by using a breathing simulator^(7–9)^ (Harvard pump, model No. 618; Harvard Respiratory Apparatus, Millis, MA) set to deliver a slow and deep sinusoidal breathing pattern (1.5 L, 6 breaths/min, duty cycle of 0.7) (Fig. 2). The long duty cycle (inspiratory time/total time of the breath) provides an average inspiratory time of ∼6–7 seconds with a short expiratory time. This pattern of breathing is similar to a breath-hold, designed to maximize deposition and minimize expiratory losses. The same pattern was used in the Diaz study facilitated by the Philips I-neb device, which also incorporated an inspiratory resistance. Filters (Pari, Starnberg, Germany) were placed between the nebulizer and the respirator and on the expiratory port of the mouthpiece: the former, to capture all inhaled particles that would be presented to a patient (Inhaled Mass); the latter, particles generated during the expiratory phase of breathing plus particles in the tubing dead space (Expiratory Phase Filter). The nebulizer was run to “dryness” determined by direct observation in approximately 10 minutes. The nebulizer charge consisted of 2 mL saline mixed with 300 μCi ^99m^Technetium bound to diethylenetriaminepentaacetic acid (DTPA).^(10)^ To measure particle size distribution, a cascade impactor (Marple 8-stage impactor, 2 L/min flow; Thermo Fischer Scientific, Waltham, MA) was placed between the nebulizer and the breathing simulator. Percent activity per cascade stage was plotted as log aerodynamic diameter versus probability.

### Clinical protocol

Thirteen individuals participated in this study: four healthy volunteers and nine volunteers with IPF (Table 1). All volunteers signed an IRB-approved consent form. Patient volunteers were diagnosed in our interstitial lung disease clinic. Of the nine patients with IPF, three were on oxygen. The patient volunteers had moderate to severe restrictive lung disease as defined by the American Thoracic Society/European Respiratory Society consensus guidelines with pulmonary function values similar to those reported in clinical trials for systemic drugs^(2–4)^ and the subjects treated in the study by Diaz et al.^(5)^ As shown in Table 1, Diffusion Capacity (diffusion capacity of lungs for carbon monoxide [DLCO]) was significantly reduced with enhanced forced expiratory volume in 1 second (FEV_1_)/forced vital capacity (FVC) ratios consistent with increased elastic recoil.

**Table 1.** Pulmonary Function: Normal and Idiopathic Pulmonary Fibrosis Subjects (% Predicted)

	DLCO %	FEV_1_ %	FVC %	FEV_1_/FVC^a^
Normal				
1	95	84	81	79
2	93	103	99	77
3	96	99	105	74
4	91	115	120	69
Mean	93.8	100	101	74.8
SEM	1.11	6.39	8.07	2.17
IPF				
1	54	129	105	89
2	65	104	89	81
3^b^	50	68	57	89
4^b^	47	114	96	82
5	37	65	54	82
6	57	117	96	82
7^b^	37	83	72	88
8	44	67	64	74
Mean	48.9	93.4	79.1	83.4
SEM	3.44	9.08	6.99	1.81
*p*^c^	0.0040	0.8081	0.0768	0.0162

^a^ FEV_1_/FVC FEV_1_ as a % FVC.

^b^ On oxygen.

^c^ Mann**–**Whitney test comparing normal subjects with IPF subjects.

DLCO, diffusion capacity of lungs for carbon monoxide; FEV_1_, forced expiratory volume in 1 second; FVC, forced vital capacity; IPF, idiopathic pulmonary fibrosis; SEM, standard error of the mean.

The actual quantity of aerosol particles deposited in the patient was measured indirectly by using a “mass balance.” That is, aerosol particles are mixed with a soluble form of radioactivity. In previous studies, it has been shown that radiolabeled saline can serve as a drug substitute that can quantify device behavior before actual clinical trials.^(9,11–14)^ The initial amount of radioactivity placed in the nebulizer was measured in a well counter and, after inhalation, all the available radioactivity was measured that remained in the nebulizer, was exhaled into the filter, and deposited on the nebulizer connector and tubing. Therefore, the mass balance technique captured all nebulized radioactivity except that which actually deposited in the patient, specifically, nebulizer residual, expiratory phase filter, tubing, and mouthpiece. The sites of deposition in the lungs and upper airways were determined by a gamma camera.

To partition deposited radioactivity between upper airways and lungs, any visible oropharyngeal activity was washed into the stomach with a drink of water and the stomach was imaged along with the lungs. Using regions of interest, stomach activity was determined from stomach counts by using a separate measure of stomach attenuation. With stomach activity quantified, the remaining deposited activity defined lung deposition. This technique of mass balance has been used by our group in many studies and has been shown to be equivalent to other indirect measures of lung deposition that measure lung attenuation separately such as by perfusion scanning.^(13,15–19)^

Each individual sat in front of a computer-controlled gamma camera (Maxi Camera 400; General Electric, Horsholm, Denmark; Model 604/150/D; Power Computing, Austin, TX; Nuclear MAC, version 4.2.2; Scientific Imaging, Inc.), and a 15-minute ^99m^Tc room background was obtained. The lung outline and regional lung volume was assessed by transmission imaging as described by Zeman et al.^(20)^ To develop a transmission image, a double-walled Lucite container with a circular space between the walls was filled with water containing 2 mC of ^99m^Tc and placed in front of each subject ∼15” from the gamma camera. In two minutes, static images were obtained.

For aerosol inhalation, each patient was given a test InspiRx Mini nebulizer containing 2 mL normal saline and coached to inhale slow and deep with relaxed breathing. The flow limiting orifice resulted in controlled inspiration taking ∼6 seconds to inspire slowly to near total lung capacity (TLC) and then exhale comfortably to below functional residual capacity (FRC). In 3–5 minutes, subjects established a steady breathing pattern. Once the training program was concluded, subjects were given a pretested Mini neb (one of the five previously bench tested devices) filled with 2 mL of normal saline radiolabeled with ∼200 μCi ^99m^Tc DTPA. The filled nebulizer was measured in a well counter (Atomlab 100; Biodex Medical Systems, Inc., Upton, NY). The nebulizer was equipped with a standard mouthpiece fitted with an exhalation valve and a Pari filter to capture all particles exhaled by the patient and generated during expiration (Expiratory Phase Filter). After the nebulizer was run to dryness, in ∼10 minutes, the patient was given a glass of water to wash oropharyngeal contents into the stomach. Immediately after completion of the treatment, a deposition scan encompassing both the lungs and stomach provided lung and upper airway (stomach activity) regional deposition. Remaining activity in the nebulizer was measured in the well counter. Activity on the filter, mouthpiece, and tubing was measured on the gamma camera after calibration with a known source.

After completion of the deposition imaging, those subjects with measurable stomach activity were restudied to measure stomach attenuation. After 15–30 minutes, a repeat image was obtained to serve as a new background and each subject swallowed a measured amount (∼250 μCi) ^99m^Tc-DTPA absorbed on a small cracker with water. A static image of the stomach was acquired, which allowed for measurement of stomach attenuation (AC_stomach_).^(21)^

### Analysis

#### Mass balance

The mass balance technique captures all nebulized radioactivity except that which actually deposits in the patient specifically, nebulizer residual, expiratory phase filter, tubing, and mouthpiece. Therefore,

$$Deposition_{patient}\left(\mu Ci\right)=nebulizercharge\left(\mu Ci\right)-\left(nebulizerresidual\left[\mu Ci\right]+expphasefilter\left[\mu Ci\right]+tubingandmouthpiece\left[\mu Ci\right]\right)$$

Stomach deposition is calculated from regional stomach counts (CPM) and the separately measured stomach attenuation (AC)_stomach_ in CPM/μCi, where CPM is counts/min from the stomach region,^(21)^ for example

$$Deposition_{stomach}\left(\mu Ci\right)=CPM\left(fromstomachregion\right)∕{\left(AC\right)}_{stomach}$$

$$Deposition_{Lung}=Deposition_{patient}-Deposition_{stomach}$$

Deposition was reported as percent of nebulizer charge to allow comparison between patients.

Regional deposition calculations were performed by using a computer as described in Diaz et al. Whole lung and central regions were hand drawn around the transmission images. Central regions encompassed 30% of the total lung outline. Regional central to peripheral ratios for deposition were normalized by the transmission regional activity providing an estimate of deposition per unit volume as outlined by Zeman et al. [e.g., normalized central to peripheral ratio (nC/P) ratio].^(20)^ Data were reported as mean ± standard error, and group comparisons were made by using the Mann**–**Whitney test.

## Results

**FIG. 3.**
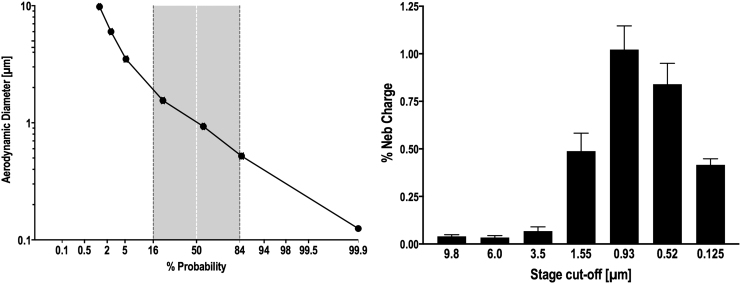
Particle distribution measured via cascade impaction. Left; ordinate: log particle size, abscissa: probability. Right; bar graph of cascade stage activity: ordinate: % nebulizer charge, abscissa: stage cutoff diameter (μm). Standard error bars are shown (*n* = 5).

The bench studies resulted in an inhaled mass of 41.8% ± 2.19 (Table 2), with 7.8% ± 0.34 captured on the expiratory phase filter representing particles generated during expiration and in tubing dead space. Nebulizer residual averaged 51.9% ± 2.16. Cascade impaction data indicated that the mass median aerodynamic diameter (geometric standard deviation) averaged 1.04 (1.92) μm (Fig. 3).

**Table 2.** *In Vitro* Characterization Studies (Mass Balance, % Nebulizer Charge)

Neb	IM %	Expiratory phase filter %	Tubing %	Nebulizer residual %	Mass balance %	MMAD (μm)
1	40.9	6.5	0.3	55.6	103	1.0
2	34.2	8.0	0.4	58.0	101	1.0
3	44.9	8.0	0.5	48.2	102	1.1
4	47.1	8.5	0.5	46.6	104	1.1
5	41.9	7.8	0.3	51.0	101	1.0
Mean	41.8	7.8	0.4	51.9	102	1.04
SEM	2.19	0.34	0.045	2.16	0.58	0.03

Expiratory Phase Filter measures aerosol generated by the nebulizer during expiration.

IM, inhaled mass.

**FIG. 4.**
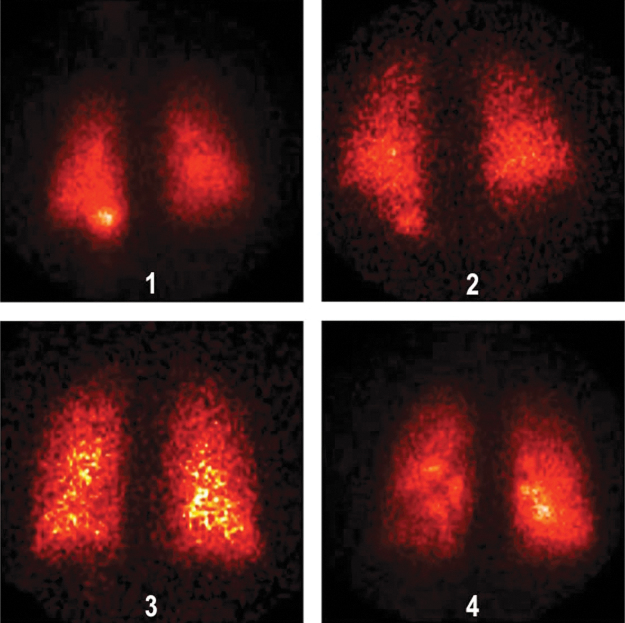
Deposition images for normal subjects.

Deposition images are shown in Figures 4 (normal subjects) and 5 (patients with IPF). *In vivo* mass balance data are listed in Table 3. Percentages refer to the nebulizer charge. The emitted dose inhaled by the patients, for example, aerosol actually leaving the nebulizer tubing was 48% (100 − [residual + tubing]), similar to the inhaled mass from the bench studies. Approximately half of the inhaled particles deposited in the lungs. *In vivo* lung deposition averaged 26.2% ± 1.83 in healthy volunteers and 23.4% ± 1.60 in IPF volunteers. Upper airway deposition, defined as stomach deposition, averaged 1.4% ± 0.83 and 2.3% ± 0.48, respectively. Regional lung deposition, quantified by the ratio of central to peripheral distribution of deposited particles normalized for volume (nC/P ratio), averaged 1.32 ± 0.050, in healthy volunteers and 1.28 ± 0.046, *p* = 0.912 in IPF volunteers, indicating a peripheral distribution in the lungs.

**FIG. 5.**
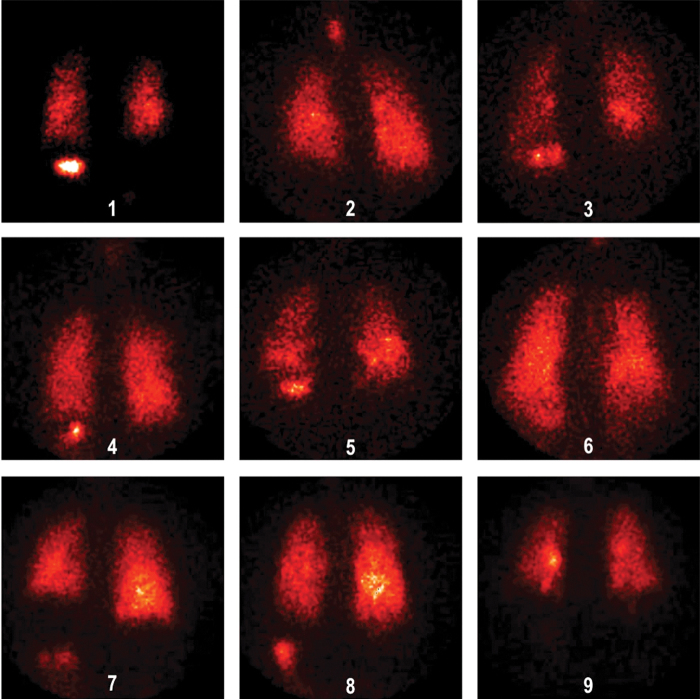
Deposition images for IPF patients. IPF, idiopathic pulmonary fibrosis.

**Table 3.** Deposition for Normal and Idiopathic Pulmonary Fibrosis Subjects (% Nebulizer Charge) and Regional Distribution in Lungs

	Lung deposition %	Stomach deposition %	Neb residual %	Expiratory phase filter %	Mouthpiece+tubing %	Ti	aC/P	nC/P^a^
Normal								
1	29.4	3.3	49.1	17.6	0.6	6	0.9	1.46
2	26.4	2.2	54.7	14.7	2.0	6	0.81	1.24
3	21.1	0	50.3	28.2	0.4	6	0.74	1.26
4	26.0	0.3	52.3	21.1	0.3	6	0.82	1.32
Mean	25.7	1.45	51.6	20.4	0.825	6	0.818	1.32
SEM	1.72	0.786	1.23	2.91	0.397	0	0.0328	0.0479
IPF								
1	29.2	4	46.8	19.7	0.3	6	0.64	1.08
2	21.6	0.8	56.7	20.8	0.1	5.5	0.86	1.33
3	15.1	1.2	61.6	21.8	0.3	4	0.79	1.4
4	23.2	2.8	55.4	18.4	0.2	5.5	0.79	1.29
5	18.8	1.9	58.5	20.6	0.2	3	0.6	1.19
6	26.6	3.3	53.2	16.5	0.4	6	0.69	1.08
7	23.3	4.2	50.2	21.7	0.6	4	0.76	1.35
8	30.0	2.8	50.2	16.1	0.9	5	0.93	1.4
9	23.9	0	53.2	22.2	0.7	6	0.97	1.44
Mean	23.5	2.33	54	19.8	0.425	5	0.781	1.28
SEM	1.59	0.484	1.53	0.76	0.0996	0.363	0.0418	0.0456
*p*^b^	0.6042	0.386	0.3958	0.9399	0.3203	0.0769	0.5776	0.9119

AC = stomach attenuation correction (CPM/μCi); lung deposition = 100 − (stomach deposition + expiratory phase filter + Neb residual + tubing-mouthpiece); stomach deposition = CPM in stomach region/AC.

^a^ Values normalized by Transmission image.

^b^ Mann-Whitney between groups.

## Discussion

This study demonstrates that it is possible to control drug delivery to the deep lung by using a jet nebulizer in patients with IPF while avoiding upper airway (throat and central airways) deposition. The nebulizer mechanically controlled inspiratory time and flow, limiting inspiratory impaction and promoting deposition in small airways and alveoli via settling. For a small fill volume of only 2 mL, the inhaled mass was one of the highest reported for a jet device (∼42% measured on the bench, with an emitted dose in the patient study of 48%), producing particles significantly smaller than those typically reported from our laboratory.^(13,15,22,23)^ Using a device producing particles in the 1.0 μm range risks reduced deposition in the lung periphery due to reduced settling. For example, in normal subjects, inhaling monodisperse 1 and 2 μm particles, Messina et al. found that 95% of inhaled 1 μm particles might be exhaled during tidal breathing. In obstructed patients, the fraction that deposited increased.^(24)^ In fibrotic lungs, therefore, fine particle aerosols designed to bypass upper and central airways might be mostly exhaled. This problem was overcome by controlling inspiration, resulting in similar lung deposition between normal and diseased subjects with varying degrees of lung involvement. Other devices have delivered controlled amounts of drug to the lungs. For example, the AERx (Aradigm, Hayward, CA), an electronic breath-actuated piston-driven nebulizer, delivered fractions as high as 80% of the charge to the lungs of subjects with asthma.^(16)^ Those particles, however, were 2 μm and they were inhaled rapidly. Other technologies such as the vibrating mesh of the I-neb used by Diaz et al. reported deposition of 65% with significant upper airway deposition. Computer-controlled breath-actuated controllers such as Akita (Vectura, Chippenham, United Kingdom) are similar to I-neb but require an assigned nebulizer technology, either jet nebulizer or vibrating mesh. Because a suitable delivery system was not readily available for use in our IPF studies, we developed a modified jet nebulizer that, with adjustments to the nebulizer charge, will deposit similar doses to the lung periphery as measured by Diaz et al. They delivered 65 μg of interferon to their patients from a nebulizer charge of 100 μg. Based on the results of this study, 300 μg placed in the i-NEB mini in 2.0 mL will deposit on average 0.234 (300) or 70 μg.

Deposition studies of hand-held nebulizers are readily quantified by the mass balance technique, because all the radioactivity nebulized and deposited outside of the patient can be readily measured. We favor oropharyngeal washing for assessing upper airway activity, because the lungs and stomach can be imaged without moving the camera. Measuring stomach attenuation is easy and less invasive than other techniques for defining organ attenuation such as perfusion imaging. This technique also ensures that any activity in transit from mouth to stomach during aerosol inhalation is accounted for. We have verified the routine completeness of oropharyngeal washing in separate studies in children where oropharynx, lungs, and stomach can be visualized on one image.^(25)^

Visual inspection of the deposition images reveals scans that are consistent with an alveolar deposition pattern. Theoretically, one would expect a normalized C/P ratio of 1.0 if deposited particles were distributed peripherally proportionately to lung volume. The transmission technique of volume normalization results in C/P ratios that are systematically somewhat higher than using xenon gas and our values of nC/P are similar to those measured in subjects with xenon nC/P ratios of 1.0 from Zeman et al.^(20)^ These data suggest that in a clinical trial the device will deliver similar alveolar doses across a population of patients with a range of lung disease.

This study was limited in that only a saline formulation was tested in a limited number of patients. More studies will be needed before starting large clinical trials. Our experience with this patient population is growing. Coughing during inhalation of our interferon formulation was a significant problem reported in the study by Diaz et al., possibly due to centrally deposited particles containing mannitol. In a similar group of IPF patients inhaling mannitol only, Kanth et al. demonstrated that coughing was prevented by controlling the particle distribution using an aerosol similar to that used during this study.^(6)^ In a given patient, coughing during aerosol inhalation is always a possibility and the other required excipients in our interferon formulation may, in future studies, cause coughing; but the two most obvious factors, mannitol and particle size have been minimized.

These findings suggest that the i-NEB Mini is optimized for future studies that will test the full interferon formulation containing the active drug as well as needed excipients. This experience also demonstrates that it is reasonable to approach the treatment of IPF by using targeted aerosols, because it is possible to overcome the potential difficulties in attempting drug delivery to diseased lungs.

## Notes

Reviewed by:

P. Worth Longest

David Cipolla

William Bennett
